# DNA methylation valley as a distinguishing feature occurs in root-specific expressed nicotine-related genes in *Nicotiana attenuata*

**DOI:** 10.3389/fpls.2025.1647622

**Published:** 2025-08-05

**Authors:** Ahui Tong, Bingwu Wang, Jinsong Wu

**Affiliations:** ^1^ Yunnan Key Laboratory for Wild Plant Resources, Kunming Institute of Botany, Chinese Academy of Sciences, Kunming, China; ^2^ University of Chinese Academy of Science, Beijing, China; ^3^ Yunnan Academy of Tobacco Agriculture Sciences, Kunming, China

**Keywords:** nicotine, DNA methylation, DNA methylation valley, nicotiana, root-specific expression

## Abstract

**Introduction:**

Nicotine, the main defense alkaloid of *Nicotiana* species, is synthesized exclusively in the roots. Several studies have shown that changes in DNA methylation patterns are associated with altered expression of genes involved in the biosynthesis of some secondary metabolites. It remains unknown whether DNA methylation pattern of nicotine-related genes differs in root and leaf tissues. We performed RNA sequencing, quantitative real time PCR and whole-genome bisulfite sequencing of *Nicotiana attenuata* root and leaf samples to investigate the DNA methylation patterns of root-specific expressed nicotine-related genes.

**Results and discussion:**

We found that most of the nicotine-related genes were exclusively and highly expressed in the root, while their DNA methylation patterns were very similar in both tissues. Interestingly, these genes with root-specific expression share a prominent DNA methylation valley (DMV) as a distinguishing feature. Further analysis revealed that 37.4% of the root-preferentially expressed genes were DMV genes, suggesting that root-specific expressed genes, including these nicotine-related genes, were strongly associated with DMV. Our results revealed that having a DMV is a common feature of most nicotine-related genes that are expressed only in the root. Thus, our results provide new insights into the regulation of alkaloid biosynthesis by epigenetic modification.

## Highlight

Nicotine, the well-known defense alkaloid of *Nicotiana* species, is synthesized exclusively in the roots. Here we show that most nicotine-related genes expressed only in the root share a DNA methylation valley as a common feature.

## Introduction

Nicotine, the most abundant alkaloid in tobacco species (Saitoh et al., 1985), plays a vital role in the plant’s defense against insect herbivores (Steppuhn et al., 2004). Interestingly, nicotine is biosynthesized exclusively in the roots, rather than in the leaves where the insect attacks take place. This idea was clearly supported by reciprocal grafting experiments between tobacco and tomato over 80 years ago (Dawson, 1941, Dawson, 1942a, Dawson, 1942b). However, the mechanism behind this is still unclear.

Nicotine biosynthesis involves two independent metabolic pathways: the pyridine nucleotide cycle and the methylpyrrole pathway. These two pathways synthesize two important precursors of nicotine, nicotinic acid and putrescine. L-aspartate was converted to nicotinic acid by aspartate oxidase, quinolinate synthetase, and quinolinic acid phosphoribosyltransferase (QPT) in both root and leaf tissues (Dewey and Xie, 2013). Putrescine can be formed directly by decarboxylation of ornithine or indirectly by arginine decarboxylase-mediated decarboxylation of arginine (Dewey and Xie, 2013). The methylation of putrescine to form *N*-methylputrescine is catalyzed by putrescine methyltransferase (PMT), this reaction commits the production of the alkaloid nicotine. Consequently, PMT has been demonstrated to be a pivotal regulatory enzyme in nicotine production (Mizusaki et al., 1971, Mizusaki et al, 1973; Biastoff et al., 2009). The *N*-methylputrescine is further oxidized to *N*-methylaminobutyric acid by *N*-methylputrescine oxidase (MPO) (Mizusaki et al., 1973). Nicotine is finally formed by conjugation of the pyridine and pyrrolinding rings, probably mediated by isoflavone reductase-like protein A622 and berberine bridge enzyme-like protein (BBL) (Deboer et al., 2009; Kajikawa et al., 2009, Kajikawa et al., 2011). Many research has shown that nicotine biosynthetic enzyme genes, including *PMT*, *A622* and *BBL*, are expressed in a root specific manner (Shoji et al., 2000; Kajikawa et al., 2017).

The expression of genes involved in nicotine biosynthesis and transport is coordinately regulated by jasmonate (JA) and a number of transcription factors. Tobacco plants have been shown to accumulate JA in response to insect feeding or mechanical damage, which in turn up regulates nicotine biosynthesis (Baldwin et al., 1994). MYC2, a key transcription factor in the JA pathway, has the capacity to bind to the promoter of *PMT* and activates its transcription (Shoji and Hashimoto, 2011). NtMYB305a also positively regulate the nicotine biosynthesis and the expression of *NtPMT* and other nicotine pathway genes (Bian et al., 2022). The ethylene response factors *NtERF189* and *NtERF199* have also been reported to be specifically expressed in roots, which are the main genes responsible for nicotine biosynthesis in the *NIC2* and *NIC1* motifs, which bind to GCC-boxes (Shoji et al., 2010; Qin et al., 2021). However, how *Nicotiana* plant controls this root-specific expression of nicotine-related genes is still unknown.

In addition to nicotine, the genes responsible for the production of valuable secondary metabolites, such as artemisinin, paclitaxel, and tanshinone, also exhibit notable tissue-specific expression (Wang and Peters, 2022; Yu et al., 2020; Zhang et al., 2018). There are many mechanisms that would enable single gene tissue-specific expression, such as tissue-specific cis-regulatory elements within promoter regions, trans-regulatory roles of transcription factors, and post-transcriptional modifications (Elmayan and Tepfer, 1995; Shang et al., 2014; Zhang et al., 2021). However, the biosynthesis of secondary metabolites typically involves the co-expression of multiple catalytic genes and transcriptional regulators. These genes exhibit a highly spatiotemporal expression pattern in specific tissues. Current research on secondary metabolites, such as capsaicin and artemisinin (Chen et al., 2024; Zhou et al., 2021), has confirmed that these multigene co-regulatory networks might be mediated by mechanisms like epigenetic modifications or transcription factor complex formations.

DNA methylation is an important epigenetic modification involved in the response of plants to biotic and abiotic stresses and in the regulation of gene expression (Yong-Villalobos et al., 2015; Palomar et al., 2021; Agorio and Vera, 2007). Differential methylation in gene regions or their flanking areas may affect their expression (Candaele et al., 2014; Vining et al., 2012; Xue et al., 2022). Tanshinone was increased by 1.5~5-fold in hairy roots of *Salvia miltiorrhiza* following the treatment with the DNA methylation inhibitor 5-azacytidine (5-AZ), which is associated with the demethylation in the promoter region of the tanshinone biosynthetic gene copalyl diphosphate synthase (Yang et al., 2022). Treatment with 5-AZ significantly reduced DNA methylation levels on nicotine *N*-demethylase *CYP82E4*, thereby increasing its expression and altering the nicotine conversion phenotype (Wang et al., 2025).

Recent studies have shown the presence of hypomethylated or unmethylated regions in plant genomes called DNA methylation valleys (DMVs). Studies in soybean have shown that DMVs overlap in various regions of the seed and in different tissues. DMV genes are enriched in transcriptional regulators and other genes essential for seed development (Chen et al., 2018).

It is currently unknown whether the root-specific expression of nicotine-related genes is associated with specific DNA methylation patterns. In this study, we used RNA sequencing and whole-genome bisulfite sequencing (WGBS) technology and found that nicotine-related genes were expressed only in the root, while their DNA methylation patterns were very similar in both tissues, but shared a prominent DMV.

## Material and methods

### Plant material and DNA methylation inhibitor treatment

Seeds from the 35^th^ generation of an inbred line of *N. attenuata* were used as wild type (WT) in this study. Seed germination and plant growth were performed as described in Krügel et al. (2002).

5-azacytidine (5-AZ, Sigma) was dissolved in dimethyl sulfoxide (DMSO). *N. attenuata* seeds were sterilized and germinated on Gamborg’s B5 medium as described. After 3 d, seedlings were carefully transferred to fresh GB5 medium supplied with 5-AZ at final concentrations of 10 µM or 50 µM. As a control, seedlings were grown on agar containing 0.1% DMSO. Seedlings were maintained in a growth chamber at 24°C (16h light, 8 h dark) for a further 8 d. Then, roots were quickly harvested for gene expression analysis.

### Whole-genome bisulfite sequencing and analysis

After 35 days of growth in soil, *N. attenuata* plants were transferred to 2.5L hydroponic pots for 14 days in 16 h of light. The source-sink transition leaves and roots were harvested from 9 plants, and three of the same tissues were randomly mixed as a biological replicate. The leaf samples are designated L1, L2 and L3, whilst the root sample are designated R1, R2 and R3. Those samples were quickly collected and stored in liquid nitrogen until Whole-genome bisulfite sequencing (WGBS).

The data presented in the study are deposited in the Genome Sequence Archive at the BIG Data Center, Beijing Institute of Genomics (BIG), Chinese Academy of Sciences repository, accession number CRA08374. 

To identify differentially methylated regions (DMRs) between root and leaf tissues, using Pearson’s chi-square test (χ2) in methylKit (Akalin et al., 2012) (version: 1.7.10). The whole genome was scanned for a 200 bp window, the minimum read coverage to call a methylation status for a base was set to 4. DMRs for each sequence context (CG, CHG and CHH) were identified according to different criteria: 1) For CG, numbers of GC in each window ≥ 5, absolute value of the difference in methylation ratio ≥ 0.25, and q ≤ 0.05; 2) For CHG, numbers in a window ≥ 5, absolute value of the difference in methylation ratio ≥ 0.25, and q ≤ 0.05; 3) For CHH, numbers in a window ≥ 15, absolute value of the difference in methylation ratio ≥ 0.15, and q ≤ 0.05; 4) For all C, numbers in a window ≥ 20, absolute value of the difference in methylation ratio ≥ 0.2, and q ≤ 0.05. The genes whose upstream2kb, genebody and downstream2kb overlap with DMR were identified as DMR-associated genes.

### Identification and characterization of DNA methylation valleys

The whole genome was scanned for a 1 kb window with a step size of 200 bp, and the average methylation level of all C sites within the window was calculated. DMV windows were defined with an average methylation level of less than 5%. Overlapping DMV windows were combined. Genes with 80% of their gene body in a DMV were identified as DMV genes.

### Transcriptome sequencing and analysis

Leaf and root samples from 35 d-old rosette staged WT plants were harvested. Total RNA from three biological replicates of samples was isolated using Trizol reagent. RNA sequencing was conducted by Shanghai OE-Biotech (http://www.oebiotech.com/) with the Illumina Hiseq 2000. Sequencing was performed at 10 G depth, and mapped to the *N. attenuata* reference genome sequence (GCF_001879085.1). The relative abundance of the transcripts was measured as reads per kilo base of exon model per million mapped reads (RPKM). Differential expression between roots and leaves was performed with a cutoff of 2-fold change.

The data presented in the study are deposited in the Genome Sequence Archive at the BIG Data Center, Beijing Institute of Genomics (BIG), Chinese Academy of Sciences repository, accession number CRA08343. 

### Quantitative PCR

Quantitative PCR (qPCR) was performed on the CFX Connect qPCR instrument (Bio-Rad) using iTaq Universal SYBR Green Supermix (Bio-Rad) with specific primers (**Supplementary Table S3**) according to the manufacturer’s instructions, as described by Song et al. (2019) As the Ct values of *Actin*, *Elongation factor 1-alpha*, and *60S ribosomal protein* were at the same level in different treatments (Song and Wu, 2023). We used *Actin*, as the reference gene in this study.

### Identification of tissue-preferentially expressed genes

The identification of tissue- preferentially expressed genes was based on the FPKM values derived from root and leaf transcriptome data. Using TBtools (Chen et al., 2020), the transcriptome data of roots and leaves were analyzed to calculate the TAU index (Yanai et al., 2005). For each gene, a TAU value ranging from 0 to 1 was calculated based on its expression levels across different tissues, with higher values indicating stronger specificity. Specifically, a TAU value of 1 represents tissue-specific expression, 0.9 indicates tissue-preferential expression, and values close to 0 suggest constitutive expression. Finally, genes with TAU values ≥ 0.99 were defined as tissue- preferentially expressed genes.

## Results

### Root-specific expression of nicotine-related genes

To investigate the expression pattern of genes involved in nicotine biosynthesis and transport in *N. attenuata*, we performed RNA sequencing of the source-sink transition leaves and roots of 35-day-old plants at the rosette stage grown in hydroponic pots.

**Figure 1:**
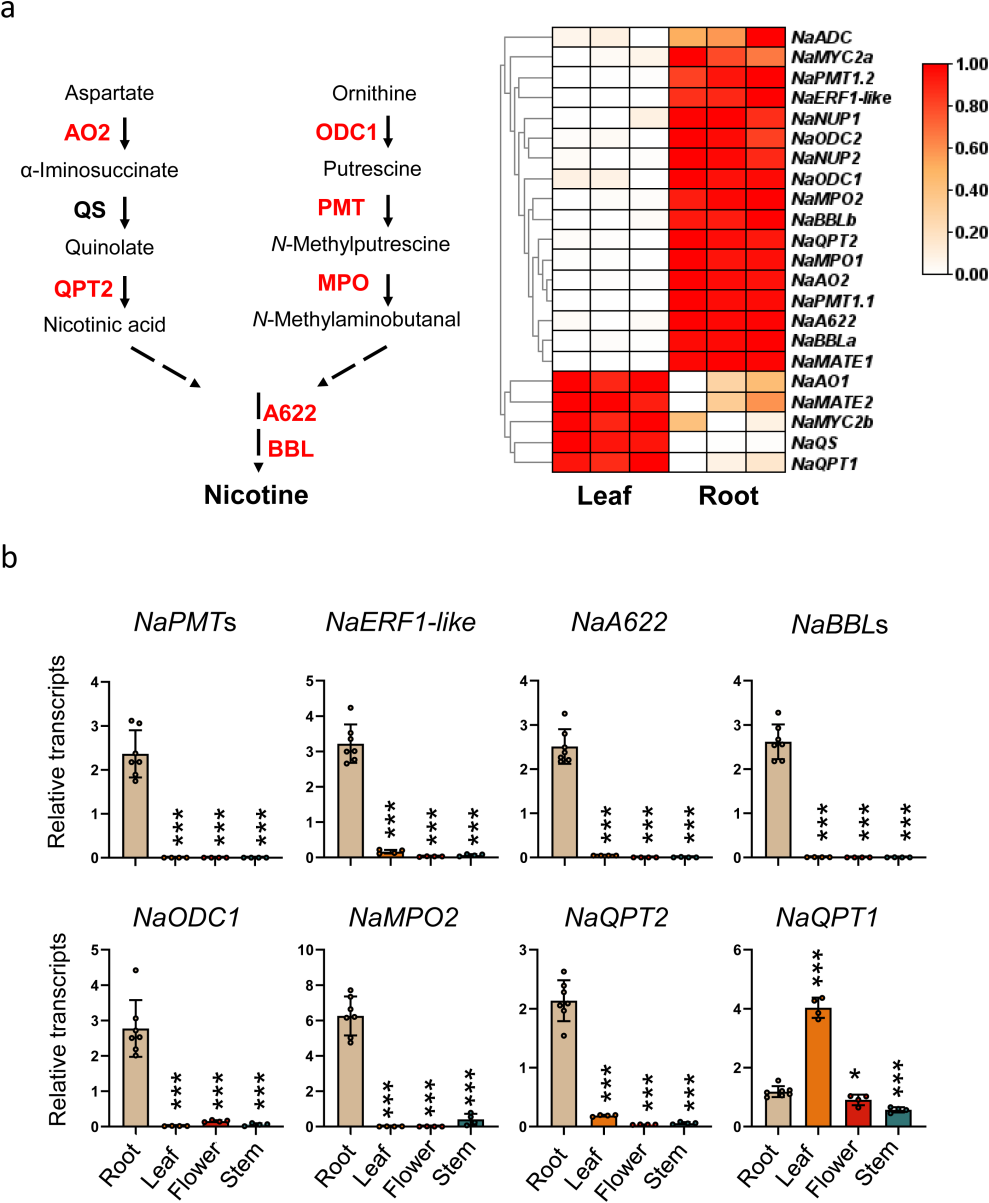
Root-specific expression of nicotine-related genes. **(a)** Schematic diagram of nicotine biosynthesis (Left panel) and heatmap showing the expression levels of nicotine-related genes in roots and source-sink transition leaves of 35-day-old *N. attenuata* plants (right panel). Each identified enzyme step is represented by an arrow and enzyme name, while a single or multi-step process that is not fully identified is represented by a dashed arrow. The enzyme names shown in red are encoded by genes with root-specific expression. ODC, ornithine decarboxylase; PMT, putrescine N-methyltransferase; MPO,N-methyltransferase oxidase; AO, aspartate oxidase; QS, quinolinate synthase; QPT, quinolinate phosphoribosyltransferase; BBL, berberine bridge enzyme-like. White and red indicate low and high transcription levels, respectively, with three replicates in each tissue. **(b)** Mean (±SD) relative transcripts of *NaPMTs, NaERF1-like, NaA622, NaBBLs, NaODC1, NaMPO2, NaQPT1*, and *NaQPT2* as measured in seven biological replicates of root, leaf, flower and stem of WT. Primer pairs detecting both *NaPMT1.1* and *NaPMT1.2* (or *NaBBLa* and *NaBBLb*) were used. Asterisks indicate significant difference between root and other tissues (Student’s t-test: *, p<0.05; ***, p<0.001).

Transcriptome analysis revealed that many nicotine-related genes are exclusively and highly expressed in roots, including nicotine biosynthesis genes, *NaAO2*, *NaQPT2*, *NaODC1*, *NaODC2*, *NaPMT1.1*, *NaPMT1.2*, *NaMPO1*, *NaBBLa*, *NaBBLb* and *NaA622*, and two genes involved in nicotine transport, *NaNUP* and *NaMATE1*, as well as the *NaERF1-like*, which is the closest homologue of *NtERF189* and *NtERF199* (**Figure 1a**). Meanwhile, *NaAO1*, *NaQS* and *NaQPT1* are more highly expressed in leaves (**Figure 1a**).

To confirm the RNA sequencing results, we measured the transcript levels of *NaPMT*s, *NaERF1-like*, *NaA622*, *NaBBL*s, *NaODC1*, *NaMPO2*, and *NaQPT2* by quantitative real time PCR (qPCR). Indeed, their transcripts were barely detectable in leaf tissue, but highly expressed in roots (**Figure 1b**). However, *NaQPT1* was highly expressed in leaves, similar to the results of transcriptome (**Figure 1b**).

### Overview of the DNA methylation patterns in the leaf and root samples

We generated single base resolution maps of DNA methylation by whole genome bisulfite sequencing (WGBS), using three biological replicates of roots and leaves. We obtained an average of 136,572,811 clean reads per sample with a Q20 quality score greater than 96.7% (**Supplementary Table 1**). The mapping ratio to the reference genome ranged from 87.71% to 94.71% (**Supplementary Table 1**). The mean coverage for whole genome methylation sequencing was 10×, approximately 80% of the cytosines were covered by at least one read (**Supplementary Figure 1b**), indicating that the data are valid and reliable for further analysis. Furthermore, Pearson’s correlation coefficients between different biological replicates were greater than 0.91 (**Supplementary Figure 1a**), indicating the high reproducibility and accuracy of our sequencing data.

**Figure 2:**
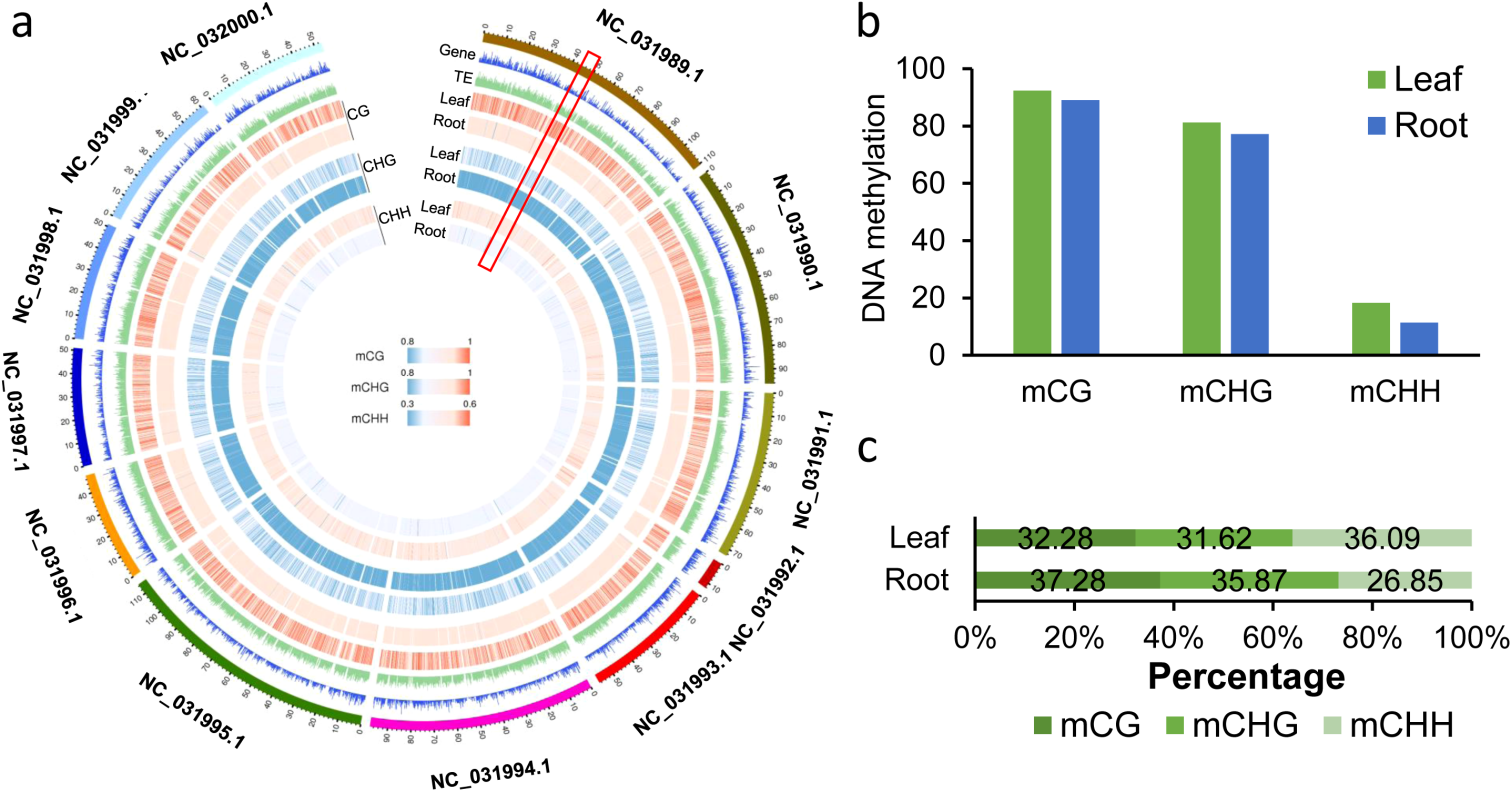
Genome-wide DNA methylation profiles of *Nicotiana attenuata* root and leaf tissues. **(a)** A circular map of CG, CHG and CHH methylation levels, genes and TE density on 12 chromosomes of *N. attenuata*. DNA methylation levels were represented by heatmaps. Blue and red indicate low and high methylation levels, respectively. Gene and TE density are represented by histograms. Average DNA methylation levels and gene/TE density were calculated using a 100 kb window. The outermost ring represents chromosomes. Followed by: gene density, TE density (TE density is the ratio of TE length to window length); CG methylation; CHG methylation; CHH methylation. For DNA methylation rings, from the outside to the inside, there are leaves and roots. **(b)** Global average DNA methylation levels of CG, CHG, and CHH in different tissues of *N. attenuata*. **(c)** Relative proportions of methyl-cytosines in the three sequence contexts in different tissues.

The genome-wide methylation of the twelve chromosomes are shown in **Figure 2a**. From the global DNA methylation distribution, we observed that the methylation levels of CG and CHG were lower in the gene-enriched regions, but higher in the transposable element (TE)-enriched regions (**Figure 2a**). These results are consistent with previous studies in other plants (Xue et al., 2022; Song et al., 2013). Interestingly, the genome-wide distribution and global DNA methylation levels varied significantly between root and leaf tissues, with the predominant methylation context being CG at 92.3% for leaves and 89% for roots. In addition, the less abundant CHH was with 18.3% in leaves and 11.47% in root (**Figure 2b**). We also found that the proportion of methylcytosine differed between roots and leaves. In leaves, the three types of DNA methylation proportions were at similar levels, being 32.28% for CG, 31.62% for CHG and 36.09% for CHH. However, the proportion of CG and CHG was higher than that of CHH, with 37.28% for CG, 35.87% for CHG and 26.85% for CHH in roots (**Figure 2c**; **Supplementary Table 1**).

### Nicotine-related genes have very similar DNA methylation patterns in the leaf and root and qualify as DMV genes

We then used the root as a control to identify differentially methylated regions (DMRs) in the leaf for three methylation contexts. Our analysis found a total of 637 CG-DMRs, 665 CHG-DMRs, and 15123 CHH-DMRs (**Supplementary Figure 1c**). The analysis showed a notable increase in CHH DMRs compared to CG and CHG DMRs. This suggests that CHH methylation exhibits greater variation between different tissues than CG and CHG methylation.

**Figure 3:**
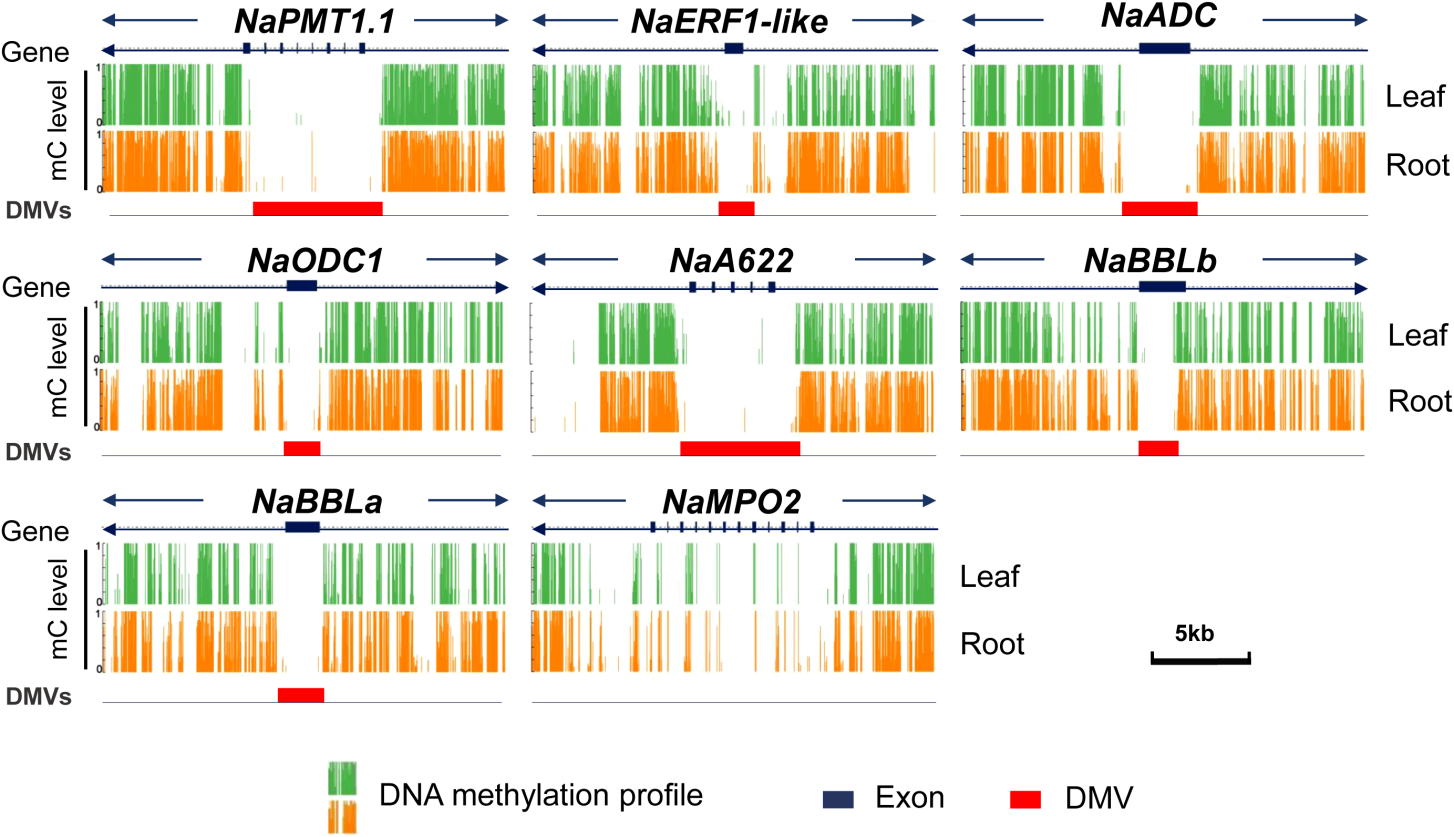
DNA methylation valley (DMV) as a distinct feature of the nicotine-related genes. The genebody and flank region (± 10 kb) of nicotine-related genes (*NaPMT1.1*, *NaERF1-like*, *NaODC1*, *NaADC*, *NaA622*, *NaBBLb*, *NaBBLa*, *and NaMPO2*) with DNA methylation patterns and DMVs as shown by integrated genomics observer (IGV). Green and orange represent DNA methylation profiles of the leaf and root, black box represents exons and red box represents DMVs.

However, no nicotine-related genes were detected in the DMRs, revealing no significant methylation differences within their gene bodies or flanking sequences. Thus, we scanned the DNA methylation patterns of all nicotine-related genes one by one in detail, including *NaPMT1.1*, *NaA622*, *NaBBLa, NaBBLb*, *NaERF1-like NaODC1* and *NaADC*. We found that all these genes have very similar DNA methylation patterns in both root and leaf samples (**Figure 3**; **Supplementary Figure 2**). Therefore, our result does not support the idea that different DNA methylation patterns are responsible for the root-specific expression of nicotine-related genes.

Interestingly, ten of sixteen nicotine genes with root-specific expression have a DNA methylation valley (DMV) as a distinguishing feature, including *NaPMT1.1*, *NaA622*, *NaBBLa, NaBBLb*, *NaERF1-like NaODC1* and *NaADC* (**Figure 3**; **Supplementary Figure 2**).

### Root-preferentially expressed genes are strongly associated with DMV

**Figure 4:**
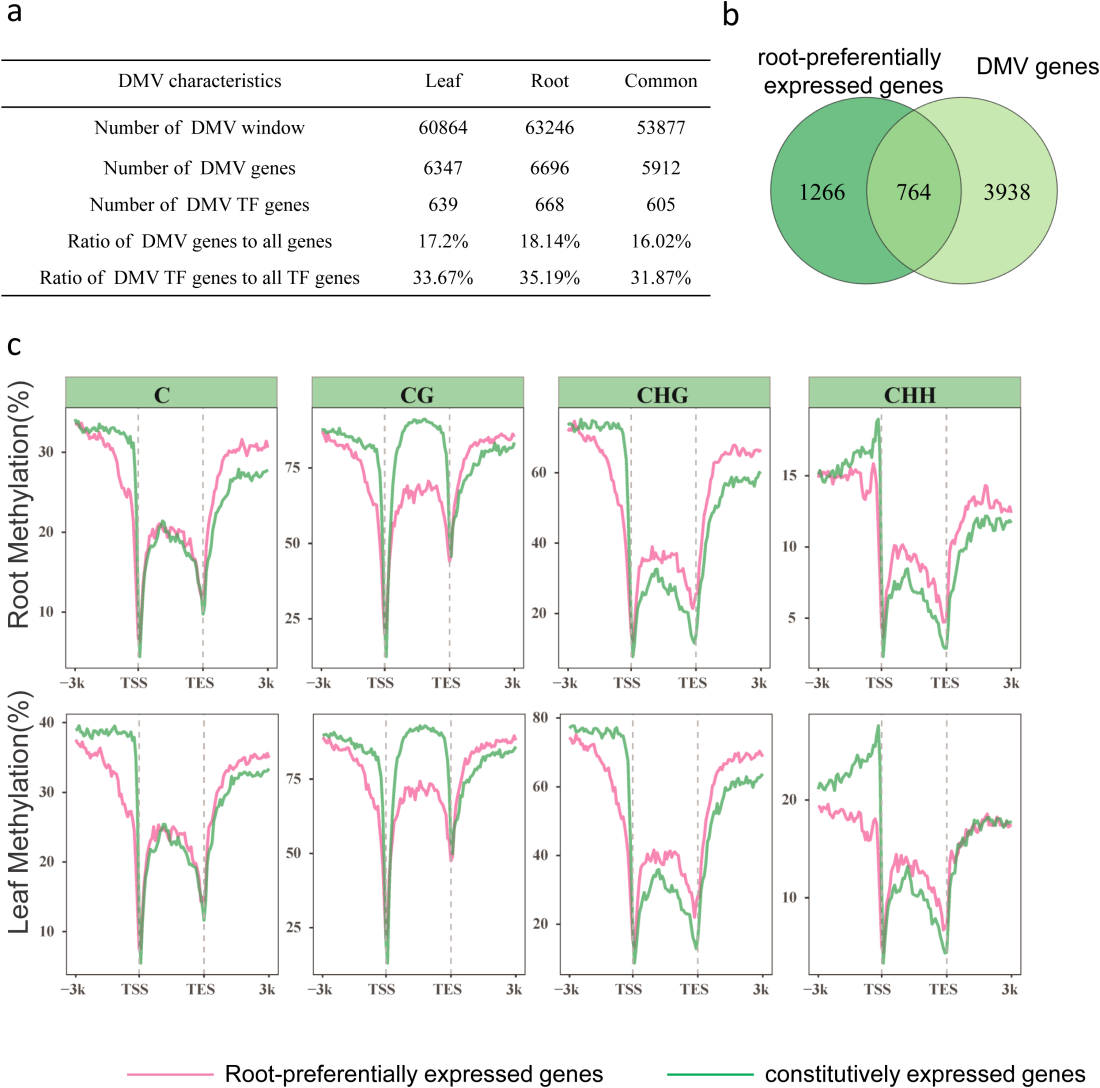
Many root-preferentially expressed genes have a DMV. **(a)** Summary of DMV windows and genes in leaf and root of *Nicotiana attenuata*. **(b)** A Venn diagram illustrates the overlap between DMV genes and root-preferentially expression genes. **(c)** Average level of genomic C, CG, CHG, CHH methylation in root-preferentially (pink line) and constitutive genes (green line) in root (up) and leaf (down). TSS, transcription start site; TES, transcription termination site.

To investigate whether DMV genes are only related to nicotine biosynthesis or not, we use 1 kb window with a step size of 200 bp, to scanned DMV windows in the whole *N. attenuata* root and leaf genome for regions with <5% bulk methylation in cytosine contexts. Overlapping DMV windows were combined. Genes with 80% of their gene body in a DMV were considered as DMV genes. We identified 6,696 and 6,347 DMV genes in roots and leaves, respectively (**Figure 4a**; **Supplementary Table 2**). Of these, 5,912 genes were common to both root and leaf samples, representing 16% of all the genes in the *N. attenuata* genome. Notably, 31% of the transcription factor genes (n =605) were found within the DMVs (**Figure 4**; **Supplementary Table 2**).

To further investigate whether these DMV genes are associated with tissue-specific expression, we analyzed the transcriptome and DMVs data together. We identified 2030 root-preferentially expressed genes, and 905 leaf-preferentially expressed genes (**Supplementary Table 3**). Among these 2935 tissue-preferentially expressed genes, we found that around 34.2% of them (1,003 genes, TAU index > 0.99) are DMV genes. Interestingly, 764 out of 2030 root-preferentially expressed genes are DMV genes (**Figure 4b**), including those genes involved in root development and morphogenesis (**Supplementary Figure 3**; **Supplementary Table 2**). The enrichment of root-preferentially expressed genes in DMV genes was verified using a hypergeometric test (*p* = <2e-16). All these data revealed that root-preferentially expressed genes are strongly associated with DMV.

We classified the transcription factors present in the DMV according to their tissue-specific expression patterns, and found that the ERF transcription factors were the most abundant. This group included NaERF1-like, which is the closest homologue of NtERF199 (**Supplementary Table 2**).

We also compared the average DNA methylation levels of root-preferentially expressed genes and genes constitutively expressed in both root and leaf tissue. For C methylation, the average methylation levels show that root-preferred genes were significantly lower than those of constitutively expressed genes near the transcription start site (TSS); at the far end of the transcription end site (TES), C methylation levels in root-preferentially expressed genes were higher than those in constitutively expressed genes. However, for the CG methylation, the root-preferentially expressed genes exhibited a significant decrease near the TSS and within the gene body regions (**Figure 4c**). Furthermore, we conducted an analysis of the mean DNA methylation levels in root-preferentially expressed genes and constitutively expressed genes across root and leaf tissues (**Supplementary Figure 4**). The analysis showed that CG and CHG methylation levels exhibited no significant difference between root and leaf tissues. However, CHH methylation levels were significantly higher in leaves compared to roots. This CHH methylation pattern was observed in both root-preferentially expressed genes and constitutively expressed genes, suggesting that these methylation differences are likely due to different tissues rather than gene-specific expression patterns

## Discussion

Nicotine, the well-known alkaloid produced by *Nicotiana* species responsible for the addictive properties of tobacco smoking, is a neurotoxin against insect herbivores. As early as 1942, it has been demonstrated that nicotine is specifically synthesized in the root and then transported to the leaves through reciprocal grafting experiments between tobacco and tomato (Dawson, 1942a, Dawson, 1942b, Dawson, 1941). As our understanding of the key enzyme genes in the nicotine biosynthesis pathway advanced, more and more molecular evidence supported this idea. Many nicotine related genes, including its key enzyme genes, *NtPMT*, *NtA622* and *NtBBL*, and key regulate genes *NtERF189*, *NtERF*199 are highly expressed in the roots, but their expression in leaves is hardly detected (Kajikawa et al., 2017). In this study, we also found that most of the nicotine-related genes are highly and specifically expressed in the root in *N. attenuata*, including *NaPMT*, *NaBBL*, *NaA622*, *NaMPO1*, and *NaERF1-like*, the homolog gene of *NtERF199* (**Figure 1**).

Several literatures have suggested that differential methylation in gene regions or their flanking areas may affect their expression (Vining et al., 2012; Xue et al., 2022; Candaele et al., 2014). Therefore, we hypothesized that the differences in DNA methylation patterns of nicotine-related genes between roots and leaves, either within gene regions or their flanking areas, might explain the preferential expression of these genes in roots. However, WGBS analysis in *N. attenuata* revealed that there were no significant differences in the DNA methylation pattern of all nicotine-related genes between roots and leaves (**Figure 3**). Thus, these results do not support the idea that DNA methylation differences in nicotine-related genes between root and leaf are responsible for their root-specific expression. Studies in soybeans have also shown that although some DNA methylation in certain regions is associated with the expression of nearby genes, the DNA methylation levels of most differentially expressed genes are similar in different organs (Song et al., 2013).

Interestingly, in both root and leaf tissues, we also found DMVs in ten of the sixteen nicotine-related genes with a root-specific expression pattern, including *NaPMT1.1*, *NaERF1-like*, *NaQPT2*, *NaA622*, and *NaBBLa NaBBLb* (**Figure 3**). Therefore, having a DMV is a common feature of most nicotine-related genes. This finding is consistent with that of Han et al. (2022) in endosperm-specific genes. In addition, our results also found that most DMV genes overlap between roots and leaves (**Figure 4a**). It is consistent with that of Chen et al. (2018), DMV regions remain stable and unmethylated across different tissues and developmental stages. Interestingly, 37.4% of the root preferentially expressed genes are DMV genes (**Figure 4b**), suggesting a strong association of root-preferentially expression and DMV.

However, the mechanisms underlying DMVs and transcriptional regulation remain unclear and unreported. DMVs are unlikely to play a direct role in transcriptional activation, as they exist even in leaves where nicotine-related genes are not expressed. Research shows that the majority of accessible chromatin regions occur within unmethylated region across many species (Crisp et al., 2020). A possible mechanism is that DMVs interact with other epigenetic modifications, such as histone modifications, to recruit chromatin remodeling factors, thereby regulating chromatin accessibility and gene expression. For instance, in castor bean, the seed-specific gene *ABI3* is activated in endosperm via histone modifications like H3K4me3, H3K36me3, H3K9ac, and H3K27ac, whereas it is repressed in leaves via H3K27me3 modification (Han et al., 2022).

We thus systematically analyzed the DNA methylation levels of all root-preferentially expressed genes and all constitutive genes. Our results showed that the average level of CG methylation shows a marked decrease within gene body regions in root-preferentially expressed genes compared to the constitutively expressed genes, a finding consistent with the presence of DMVs observed in nicotine-related genes (**Figure 4c**). Gene body methylation (GbM) is associated with moderately and constitutively expressed housekeeping genes in *A. thaliana* (Zhang et al., 2006), these data suggest that the low CG methylation in gene body region may play a role in the tissue-preferentially expression of genes.

Methylation of promoter regions is closely associated with transcriptional silencing of genes. We observed significantly lower levels of CG and non-CG methylation in the promoter regions of root-preferentially expressed genes compared to constitutively expressed genes in root tissues. Interestingly, in leaves where these genes were not expressed, the levels of CG and non-CG methylation in promoter regions were similarly significantly lower (**Figure 4c**). These results suggest that hypomethylation in promoter and gene regions is a common feature of root-preferentially expressed genes, but it does not account for their tissue-specific expression (**Figure 4c**).

Although the DNA methylation patterns are similar in roots and leaves, this suggests that they are not involved in the regulation of the tissue-specific expression of nicotine-related genes. However, it is unclear whether the expression of nicotine-related genes will be affected by DNA methylation levels in the roots. Treatment with the DNA methylation inhibitor, 5-azacytidine (5-AZ), reduced the expression of *NaA622* and *NaERF1-like*. These results suggest that altering the DNA methylation will affect the expression levels of nicotine-related genes in the roots (**Supplementary Figure 5**).

Taken all together, our results revealed that although most nicotine-related genes are expressed exclusively in roots, their DNA methylation patterns are quite similar in both root and leaf tissues. However, they have a DMV as a distinguishing feature, which is very likely related to their root-specific expression.

## Data Availability

The datasets presented in this study can be found in online repositories. The names of the repository/repositories and accession number(s) can be found in the article/**Supplementary Material**.

## Supplementary material

Supplementary Table 1 WGBS quality control, mapping ratio and mC proportion.

Supplementary Table 2 DMV genes, and their TAU index summary.

Supplementary Table 3 All genes FPKM summary.

Supplementary Table 4 All primers used in this study.
