# Supplementary material for: DNA methylation valley as a distinguishing feature occurs in root-specific expressed nicotine-related genes in Nicotiana attenuata
Source: Front Plant Sci. 2025 Aug 5;16:1647622. doi: 10.3389/fpls.2025.1647622 (PMC12361153; doi:10.3389/fpls.2025.1647622)
Supplement: Supplementary file 5 [file DataSheet1.docx]

**DNA methylation valley as a distinguishing feature occurs in root-specific expressed nicotine-related genes in *Nicotiana attenuata***

Ahui Tong^1, 2^, Bingwu Wang^3*^ and Jinsong Wu^1*^

^1^Yunnan Key Laboratory for Wild Plant Resources, Kunming Institute of Botany, Chinese Academy of Sciences, Kunming, 650201, China

^2^University of Chinese Academy of Science, Beijing 10049, China

^3^Yunnan Academy of Tobacco Agriculture Sciences, Kunming, 650021, China

*** Corresponding author**

Prof. Dr. Jinsong Wu,

Yunnan Key Laboratory for Wild Plant Resources, Kunming Institute of Botany, Chinese Academy of Sciences, Kunming, 650201, P.R. China

E-mail: [jinsongwu@mail.kib.ac.cn](mailto:jinsongwu@mail.kib.ac.cn)

**Assoc. Prof. Bingwu Wang**

Yunnan Academy of Tobacco Agriculture Sciences, Kunming, 650021, China

E-mail: bwwang76@hotmail.com

**Running title:** DNA methylation valley in nicotine-related genes

**Highlight:** Nicotine, the well-known defense alkaloid of Nicotiana species, is synthesized exclusively in the roots. Here we show that most nicotine-related genes expressed only in the root share a DNA methylation valley as a common feature.

**Supplement Figures**


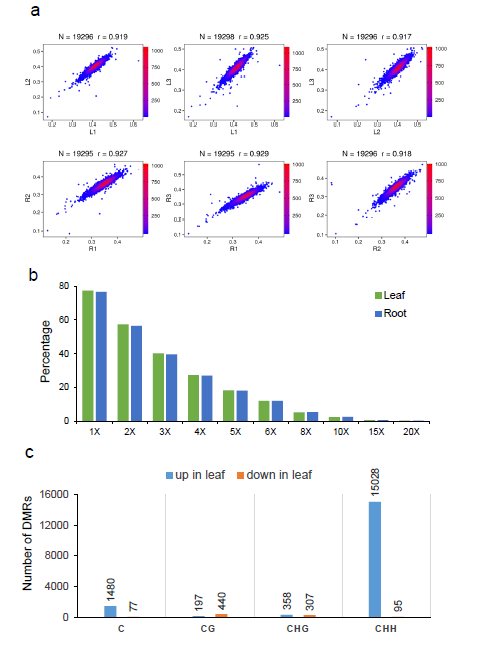


**Supplemental Fig. 1 Whole genome bisulfite sequencing (WGBS) of *N. attenuata* root and leaf samples**

**(a)** Correlation of WGBS results of leaf samples (L1, L2 and L3) and root samples (R1, R2 and R3), respectively.

**(b)** WGBS coverage was shown as the proportion of cytosines that were covered by at least ‘X’ reads

**(c)** Number of differentially methylated regions (C, CG, CHG, and CHH) as shown by bar plot by using root samples as control.


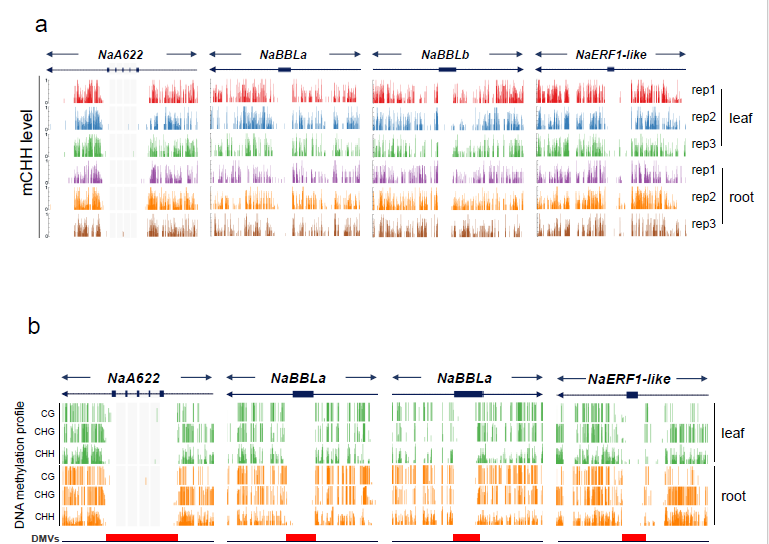


**Supplemental Fig. 2 DNA methylation valley (DMV) as a distinguishing feature of the nicotine-related genes**

**(a)** The gene body and flank region (±10 kb) of nicotine-related genes (*NaA622*, *NaBBLa*, *NaBBLb*, *and NaERF1-like*) with CHH type of DNA methylation patterns as shown by integrated genomics observer (IGV) in three biological replicates of root and leaf samples.

**(b)** The gene body and flank region (±5 kb) of nicotine-related genes (*NaA622*, *NaBBLa*, *NaBBLb*, *and NaERF1-like*,) with DNA methylation patterns and DMVs as shown by integrated genomics observer (IGV). Green and orange represent DNA methylation profiles of the leaf and root, black box represents exons and red box represents DMVs.


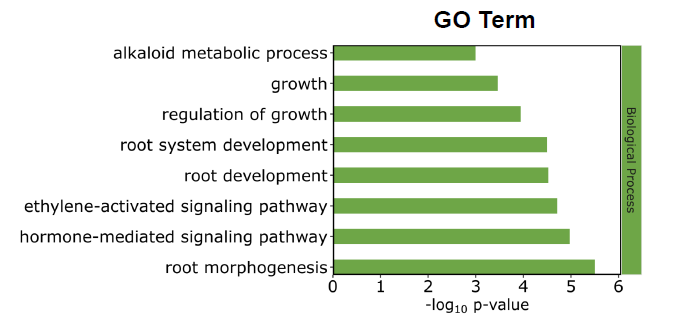


**Supplemental Fig. 3 GO enrichment of 764 root-preferentially expressed DMV genes in of *N. attenuata*.**

Many root-preferentially expressed genes, including those involved in the metabolic process of alkaloids, root development, root morphogenesis and hormone signalling, are enriched as DMV genes, like nicotine-related genes.


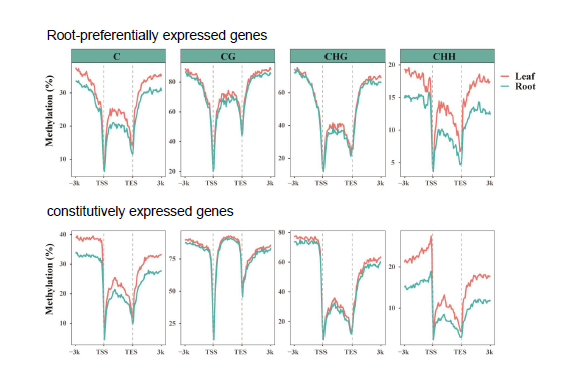


**Supplemental Fig. 4** **DNA methylation profile of root-preferentially and constitutive genes in root and leaf.**

Average level of genomic C, CG, CHG, CHH methylation of root-preferentially (up) and constitutive genes (down) in root (green line) and leaf (red line). TSS, transcription start site; TES, transcription termination site.


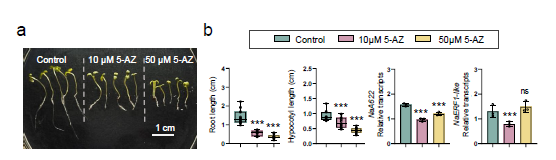


**Supplemental Fig. 5** **Treatment with DNA methylation inhibitors reduces transcription levels of nicotine-related genes in *Nicotiana attenuata*.**

**(a and b)** Root length and hypocotyl length were significantly shortened after treatments with the DNA methylation inhibitor 5-azacytidine (5-AZ) compared to the control. 0.1% DMSO was served as control. Each experimental group has at least 10 biological replicates. Box plots show the quantitative data of root length and hypocotyl length treated with 0.1% DMSO, 10 μM and 50μM 5-AZ after 8 days. Box plots indicate interquartile range and the line inside the box indicates the median. Whiskers extend to the minimum and maximum values. Asterisks indicate significant differences between treatment groups compared to control group. (Student’s t-test: ***, p<0.001; ns, no significant).
